# Lipids: fatty acids and derivatives, polyketides and isoprenoids

**DOI:** 10.3762/bjoc.13.78

**Published:** 2017-04-27

**Authors:** Jeroen S Dickschat

**Affiliations:** 1 Kekulé-Institut für Organische Chemie und Biochemie, Rheinische Friedrich-Wilhelms-Universität Bonn, Gerhard-Domagk-Straße 1, D-53121 Bonn, Germany

**Keywords:** fatty acids, isoprenoids, polyketides

Lipids fulfill various functions in life as membrane constituents, for energy storage, or as signaling molecules. If the human lipid metabolism is disturbed, this may lead to serious illnesses such as adipositas and its subsequent complications including cardiovascular diseases or diabetes mellitus. Other consequences of a disordered lipid metabolism include brain dysfunctions, especially if the sphingolipid metabolism is affected, which can often be related to a specific genetic mutation. Sensu stricto, lipids are defined as apolar natural products that can be classified as fatty acids, whose derivatives are waxes, triacylglycerides, phospholipids, sphingolipids and glycolipids. Lipids also constitute important post-translational protein modifications in lipoproteins. The amphiphilic nature of compounds such as phospholipids with a polar headgroup and a long apolar chain results in the spontaneous formation of lipid bilayers in aqueous environments. This was likely a crucial process for the origin of life – and certainly still is for all existing living systems that necessarily contain lipid membranes with their interesting and finely balanced biophysical properties. In prokaryotes, these membranes define the outer surface of individual cells, while in addition to that in eukaryotic organisms membranes are of utmost importance also for cell compartmentation, i.e., the inner structure of a cell. A large portion of these membranes is composed of steroids that influence membrane properties, such as fluidity and permeability, but have a fundamentally different biosynthetic origin from fatty acids since they are made via terpene biosynthetic pathways. Nevertheless, steroids are highly apolar yet may contain a polar headgroup such as a 3-hydroxy function (as in lanosterol). Besides membrane formation, the highly apolar character of lipids has another important consequence for signaling compounds that travel with high speed in aqueous environments – an effect that is immediately recognizable if a drop of oil is spilled on a water surface. An impressive example is reported in this Thematic Series with the use of highly apolar lactones from the African reed frog that are likely amphibian signaling compounds [1]. Sensu lato, and this is the definition relevant to this Thematic Series: lipids include all kinds of apolar (or less polar) primary and secondary metabolites, including molecules that are formed via fatty acid biosynthesis, the biosynthetically related polyketide pathways, and terpenoid biosynthesis. I hope that the present Thematic Series of the *Beilstein Journal of Organic Chemistry* on the interdisciplinary topic of “Lipids” will cover many topics of high interest to readers from chemistry, biochemistry, biophysics, medicine, pharmacy and related disciplines.

Jeroen S. Dickschat

Bonn, April 2017

## References

[1] Menke M, Peram P S, Starnberger I, Hödl W, Jongsma G F M, Blackburn D C, Rödel M-O, Vences M, Schulz S (2016). Beilstein J Org Chem.

